# Lipid Peroxidation Drives Liquid–Liquid Phase Separation and Disrupts Raft Protein Partitioning in Biological Membranes

**DOI:** 10.1021/jacs.3c10132

**Published:** 2024-01-03

**Authors:** Muthuraj Balakrishnan, Anne K. Kenworthy

**Affiliations:** † Center for Membrane and Cell Physiology, University of Virginia, Charlottesville, Virginia 22903, United States; ‡ Department of Molecular Physiology and Biological Physics, University of Virginia School of Medicine, Charlottesville, Virginia 22903, United States

## Abstract

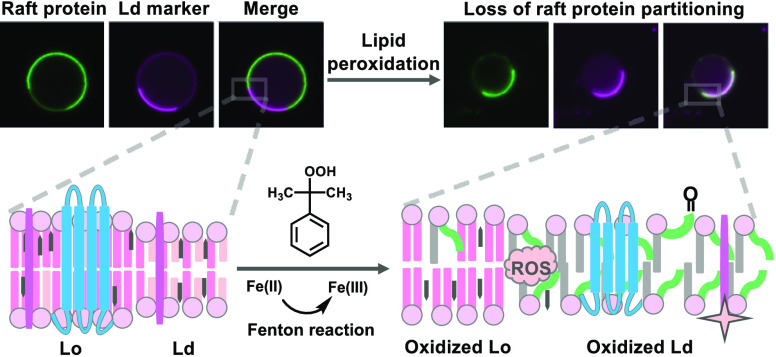

The peroxidation of membrane lipids by free radicals contributes to aging, numerous diseases, and ferroptosis, an iron-dependent form of cell death. Peroxidation changes the structure and physicochemical properties of lipids, leading to bilayer thinning, altered fluidity, and increased permeability of membranes in model systems. Whether and how lipid peroxidation impacts the lateral organization of proteins and lipids in biological membranes, however, remains poorly understood. Here, we employ cell-derived giant plasma membrane vesicles (GPMVs) as a model to investigate the impact of lipid peroxidation on ordered membrane domains, often termed membrane rafts. We show that lipid peroxidation induced by the Fenton reaction dramatically enhances the phase separation propensity of GPMVs into coexisting liquid-ordered (Lo) and liquid-disordered (Ld) domains and increases the relative abundance of the disordered phase. Peroxidation also leads to preferential accumulation of peroxidized lipids and 4-hydroxynonenal (4-HNE) adducts in the disordered phase, decreased lipid packing in both Lo and Ld domains, and translocation of multiple classes of raft proteins out of ordered domains. These findings indicate that the peroxidation of plasma membrane lipids disturbs many aspects of membrane rafts, including their stability, abundance, packing, and protein and lipid composition. We propose that these disruptions contribute to the pathological consequences of lipid peroxidation during aging and disease and thus serve as potential targets for therapeutic intervention.

## Introduction

The lipid composition of biomembranes is tuned to support the function of membrane proteins and cellular processes.^1^ Genetic defects and environmental insults that disrupt the normal repertoire of lipids can have profound consequences.^2^ One such insult is lipid peroxidation, a process driven by unregulated oxidative stress.

During lipid peroxidation, lipids containing carbon–carbon double bonds, particularly lipids containing polyunsaturated fatty acids (PUFAs) which have many such double bonds, undergo free radical attack by oxygen radicals.^3−6^ Lipid peroxidation is mediated via reactive oxygen species (ROS) and can proceed by both nonenzymatic (e.g., the Fenton reaction) and enzymatic mechanisms (e.g., lipoxygenases/oxidases). In the Fenton reaction, hydrogen peroxide (H_2_O_2_) reacts with a redox metal, such as iron, to generate a hydroxyl radical.^5,7,8^ The hydroxyl radical reacts with almost all biological molecules in living cells, forming products that can further damage biological structures. PUFA-containing lipids are particularly susceptible to this chain reaction because of their numerous carbon–carbon double bonds.^3−6^

Lipid peroxidation produces two main types of products.^3−6^ The primary products are hydroperoxide lipids. Their hydroperoxidized acyl chains become more hydrophilic and tend to associate with the lipid–water interface.^9−11^ Their presence in membranes ultimately increases membrane permeability and impacts other membrane properties such as packing, fluidity, and viscosity.^12−20^ Further oxidation generates secondary products such as reactive aldehydes, ketones, alcohols, and ethers. The best-studied secondary products of lipid peroxidation include the bioactive molecules 4-hydroxynonenal (4-HNE), a product of arachidonic acid, and 4-hydroxyhexenal (4-HHE), generated from docosahexaenoic acid. These reactive compounds form molecular adducts with lipids, proteins, DNA, and other biomolecules, thereby disrupting their normal functions.^21−23^

The biological consequences of lipid peroxidation are substantial. Lipid peroxidation triggers ferroptosis, an iron-dependent form of cell death.^24−26^ It also contributes to aging, neurodegenerative diseases such as Alzheimer’s and Parkinson’s disease, and cancer.^27−31^ To combat the deleterious effects of lipid peroxidation, efforts are underway to develop and optimize inhibitors of the process.^32−34^ Conversely, lipid peroxidation can be harnessed and exploited as a tool in photodynamic therapy to treat cancer and other diseases.^35,36^ Given these important roles of lipid peroxidation in biology, there is a substantial need to not only understand the biochemical mechanisms that underlie the process but also uncover how lipid peroxidation impacts the structure and function of cells.

Phase separation plays an important role in the function of biological systems.^37−41^ For many years, the plasma membrane of cells has been proposed to be capable of undergoing a form of liquid–liquid phase separation similar to that observed in ternary lipid mixtures consisting of a saturated lipid with a high melting temperature, an unsaturated lipid with a low melting temperature, and cholesterol, resulting in the formation of coexisting liquid-ordered (Lo) and liquid-disordered (Ld) domains.^39,42−44^ In cells, raft domains are transient and dynamic, enriched in sphingolipids, cholesterol, and lipids with saturated acyl chains, and contain a subset of membrane proteins, whereas non-raft domains are enriched in unsaturated and polyunsaturated lipids as well as membrane proteins that prefer to reside in a disordered environment.^45−49^ Rafts have been implicated in a wide range of cellular functions such as cell signaling, membrane trafficking, cellular adhesion and motility, and other essential cellular functions.^45,46,48^ There is thus considerable interest in understanding their contributions to health and disease.^46,50−55^

In model membrane systems, the presence of oxidized lipids promotes liquid–liquid phase separation.^56−60^ Furthermore, the main target of lipid peroxidation, polyunsaturated lipids, are known to regulate raft formation and composition.^49,61−70^ Yet, how lipid peroxidation impacts the formation, properties, and function of rafts in biological membranes has yet to be investigated. Here, we show that lipid peroxidation has profound consequences on the stability, abundance, packing, and protein and lipid composition of both raft-like and non-raft domains in biological membranes.

## Results

### GPMVs Are a Useful Model to Study Lipid Peroxidation

Current models suggest the lipid composition of the plasma membrane of mammalian cells is tuned to position the lipids close to a miscibility critical point, which enables them to undergo phase separation to form coexisting ordered and disordered domains in response to small perturbations.^44,48,71^ Under physiological conditions, these domains are nanoscopic in size.^46^ This makes them difficult to detect in living plasma membranes. We therefore turned to giant plasma membrane vesicles (GPMVs), a widely used model to investigate the phase behavior of biological membranes.^72−76^ Unlike in the plasma membrane of live cells, in cell-derived GPMVs, coexisting ordered and disordered domains are large enough to be directly visualized by fluorescence microscopy.^72−76^ Formation of these two macroscopic phases occurs below the miscibility transition temperature and is thought to reflect the presence of much smaller domains at physiological temperatures.^71,77,78^

GPMVs are generated by treating mammalian cells with combinations of blebbing reagents, leading to their release from the cell surface. They can then be harvested, subjected to various treatments, and incubated with fluorescent dyes to selectively label either ordered or disordered domains or probe their biophysical properties.^75,76^ For our studies, GPMVs were isolated from HeLa cells. At room temperature, a major fraction of HeLa-cell-derived GPMVs typically exhibit phase separation.^79−83^ This makes them a useful system to investigate factors that influence membranes’ liquid–liquid phase separation and the affinity of proteins and lipids for ordered versus disordered membrane domains.^72−76,84,85^

**Figure 1:**
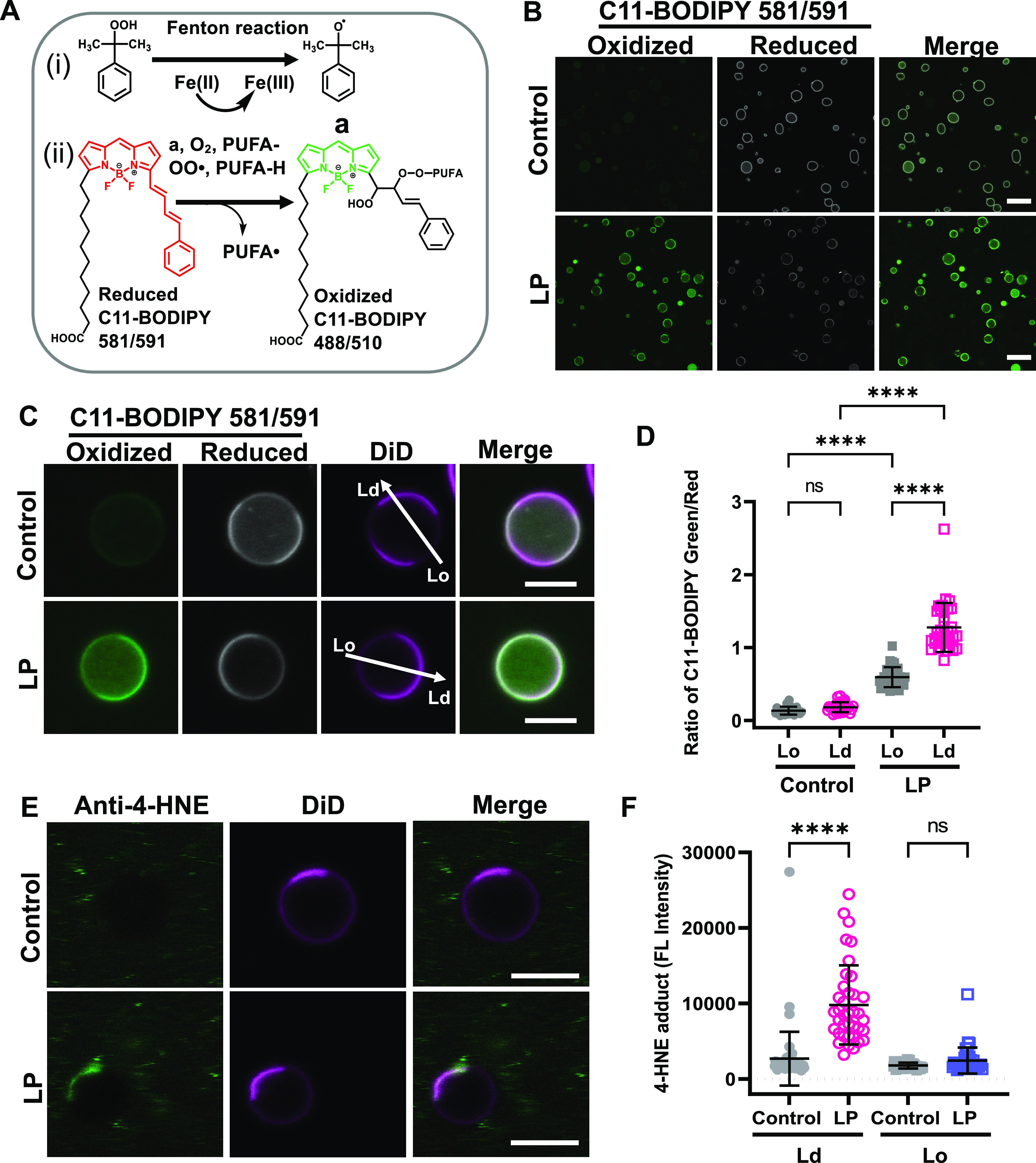
Induction and detection of lipid peroxidation in GPMVs. (A) (i) Cumene hydroperoxide in the presence of transition metal iron Fe(II) produces cumoxyl radicals via the Fenton reaction (a). (ii) Cumoxyl radicals (a) abstract a hydrogen (H) from a polyunsaturated lipid (PUFA-H) generating a lipid radical (PUFA^•^) that reacts immediately with oxygen, generating PUFA peroxy radicals (PUFA-OO^•^). The PUFA peroxy radicals react with C11-BODIPY 581/591, causing deconjugation of C11-BODIPY and a blue shift of the emission wavelength to 510 nm. (B) GPMVs were left untreated or incubated with 50 μM Fe(II) and 500 μM cumene hydroperoxide to induce lipid peroxidation, labeled with 1 μM C11-BODIPY 581/591, allowed to settle for 15–20 min, and imaged at RT using confocal microscopy. Scale bars, 20 μm. (C) Representative images of individual GPMVs labeled with C11-BODIPY 581/591 under control conditions or following lipid peroxidation. GPMVs were co-labeled with DiD to mark the position of the disordered domains. The arrows show examples of the position of lines used to analyze the fluorescence intensity in Lo and Ld domains. Scale bars, 5 μm. (D) Ratio of green (oxidized): red (reduced) BODIPY 581/591 fluorescence in ordered versus disordered domains under control conditions and following lipid peroxidation. Each data point corresponds to an individual GPMV. Error bars show mean ± SD for 27–33 GPMVs. (E) GPMVs were either left untreated or subjected to lipid peroxidation, immunolabeled with an anti-4-HNE antibody and Alexa-488 secondary antibody, and then stained using DiD. Examples of representative GPMVs are shown. Scale bars, 5 μm. (F) Quantification of immunostaining of 4-HNE levels. Fluorescence intensity is reported in arbitrary units. Each data point corresponds to an individual GPMV. Error bars show mean ± SD. P values were determined by unpaired one-way ANOVA with Sidak’s multiple comparison test, α = 0.05 (95% confidence level), ****, *P* < 0.0001; n.s., not significant. Data in (D, F) were pooled across 2 independent experiments.

To induce lipid peroxidation in GPMVs, we applied a chemical approach based on the Fenton reaction. In this reaction, cations of a transition metal such as iron interact with hydrogen peroxide to form a hydroxyl radical.^8,86^ Here, we utilized Fe(II) as an electron source and cumene hydroperoxide as a stable, lipophilic oxidizing agent^87−89^ (Figure 1A). In this reaction, the reduced form of Fe(II) is oxidized to Fe(III) by the Fenton reaction with cumene hydroperoxide to form lipophilic cumoxyl radicals. Cumoxyl radicals can also react with other cumene hydroperoxide molecules to yield cumoperoxyl radicals, which in turn leads to lipid peroxidation (reviewed in ref (23)). We chose to use a 1:10 metal/cumene hydroperoxide ratio based on a previous study.^90^

To detect lipid peroxidation, we used the lipid peroxidation sensor C11-BODIPY 581/591 (C11-BODIPY).^89,91^ C11-BODIPY binds membranes and exhibits a fluorescence emission peak shift from 590 nm in its reduced state (red) to 510 nm in its oxidized state (green) induced by lipid peroxidation (Figure 1A). In control GPMVs labeled with C11-BODIPY, primarily red fluorescence was observed (Figures 1B and S1A). In contrast, after incubating GPMVs with Fe(II) (50 μM) and cumene hydroperoxide (500 μM), the fluorescence signal from the reduced form of C11-BODIPY (red) decreased and the signal from the oxidized form of C11-BODIPY (green) concomitantly increased (Figure 1B). No fluorescence was observed in GPMVs incubated with lipid peroxidation reagents in the absence of C11-BODIPY (Figure S1). Furthermore, when either Fe(II) or cumene hydroperoxide was omitted from the reaction, C11-BODIPY remained in a reduced state, indicating that both Fe(II) and cumene hydroperoxide are required to drive lipid peroxidation (Figure S1). These results suggest that GPMVs can be used to investigate the effects of lipid peroxidation on cell plasma membranes.

In GPMVs subjected to lipid peroxidation, C11-BODIPY fluorescence tended to be enriched in one domain in phase-separated GPMVs (Figure 1C). To determine whether this partitioning corresponds to the ordered or disordered domain, GPMVs were labeled with both C11-BODIPY and the disordered phase marker DiD^92,93^ prior to imaging. In both control GPMVs and GPMVs that had undergone lipid peroxidation, the phase containing reduced C11-BODIPY was depleted in DiD fluorescence, suggesting that it corresponds to the ordered phase. Areas with high levels of green fluorescence (oxidized C11-BODIPY), on the other hand, typically also contained more intense DiD fluorescence, implying they correspond to disordered domains (Figures 1C and S2). Quantification of the ratio of green (oxidized)/red (reduced) C11-BODIPY fluorescence in Lo and Ld domains in populations of GPMVs revealed a significantly higher ratio of green versus red C11-BODIPY fluorescence in GPMVs subjected to lipid peroxidation relative to controls (Figure 1D). Interestingly, the green/red ratio was significantly higher in the disordered domains in treated GPMVs (Figure 1D). This suggests that levels of peroxidized lipids are higher in disordered than ordered domains.

Lipid peroxidation generates a variety of aldehyde products that can covalently modify proteins and lipids.^21,22^ To test whether these products were being formed under the conditions of our experiments, we examined the distribution of aldehyde 4-hydroxynonenal (4-HNE), one of the best-studied bioactive products of lipid peroxidation.^23,30^ Immunostaining of GPMVs showed that 4-HNE products were present after, but not before, lipid peroxidation (Figures 1E,F and S3). Notably, 4-HNE staining co-localized with the disordered phase marker DiD, similar to the distribution of peroxidized lipids (Figure 1E,F).

Together, these results suggest that the Fenton reaction drives lipid peroxidation in GPMVs as reported by C11-BODIPY oxidation and the presence of 4-HNE, and that these products preferentially accumulate in Ld domains.

### Lipid Peroxidation Enhances Phase Separation in GPMVs

**Figure 2:**
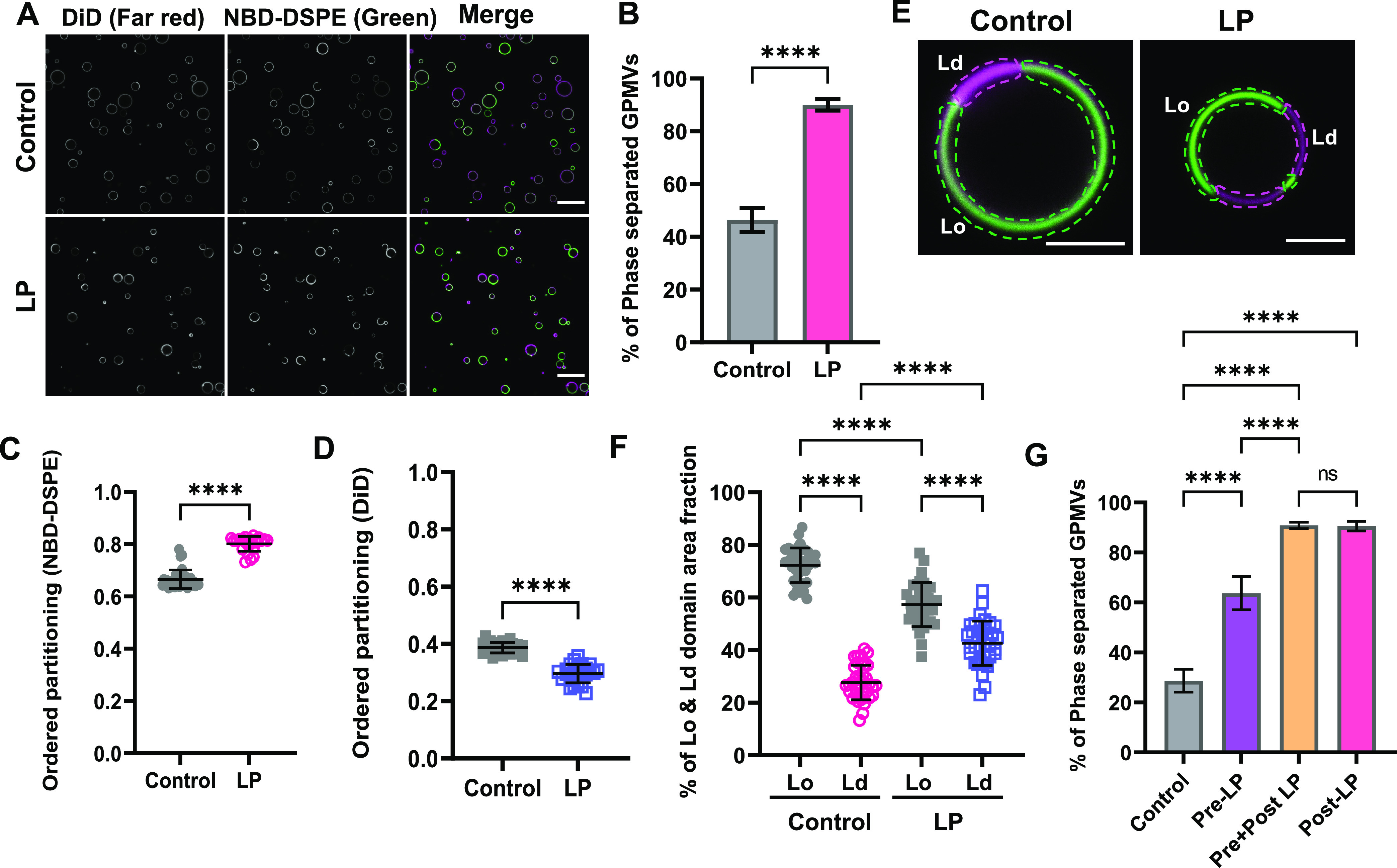
Lipid peroxidation increases the percentage of phase-separated vesicles and the area fraction of disordered domains. (A) GPMVs were either left untreated (control) or subjected to lipid peroxidation (LP). They were then labeled sequentially with NBD-DSPE (green) and DiD (magenta) prior to imaging at RT using confocal microscopy. Scale bar: 20 μm. (B) Quantification of the percentage of phase-separated GPMVs for control versus lipid peroxidation conditions. The % of phase-separated GPMVs was calculated using the green channel using VesA software. Data are presented as mean ± SD for >1000 GPMVs per group. Data were pooled across 8 independent experiments. (C, D) Impact of lipid peroxidation on ordered partitioning of Lo (NBD-DSPE) and Ld (DiD) reporter dyes. Points in (C, D) represent >100 GPMVs in each group. ****, *P* < 0.0001 using unpaired two-tailed *t* test. Data are representative of 8 independent experiments. Each data point is for an individual field of GPMVs containing >50 GPMVs. (E) Illustration of how the area fraction of ordered (green) and disordered (magenta) domains was quantified for representative GPMVs. Scale bars: 5 μm. (F) Effect of lipid peroxidation on the area fraction of Lo and Ld domains. Each data point corresponds to an individual GPMV. Bars show the mean ± SD for two independent experiments. *P* values were determined by unpaired one-way ANOVA with Sidak’s multiple comparison test, α = 0.05 (95% confidence level). ****, *P* < 0.0001. (G) HeLa cells were left untreated (control) or pretreated with lipid peroxidation reagents for 30 min at RT (pre-LP) prior to GPMV preparation. For comparison, a population of control GPMVs and pre-LP GPMVs were subsequently incubated with lipid peroxidation reagents (post LP and pre + post LP, respectively). All GPMVs were then labeled with NBD-DSPE and DiD and imaged using confocal microscopy. The % of phase-separated GPMVs was calculated using the green channel using VesA software. Data are presented as mean ± SD. *P* values were determined by unpaired one-way ANOVA with Sidak’s multiple comparison test, α = 0.05 (95% confidence level) ****, *P* < 0.0001; n.s., not significant. Data are representative of 3 independent experiments for >100 GPMVs per group.

The propensity of GPMVs to phase-separate into coexisting ordered and disordered domains depends on membrane lipid composition as well as the physicochemical properties of each phase such as their lipid packing.^68,74^ We thus next assessed the effects of lipid peroxidation on phase separation. GPMVs were labeled with NBD-DSPE (Lo domain marker) and DiD (Ld domain marker) to visualize ordered and disordered domains simultaneously.^72,82^ In control experiments, approximately 40% of vesicles exhibited phase separation (Figure 2A,B). Remarkably, after lipid peroxidation, almost all vesicles (>90%) were phase-separated (Figure 2A,B). Similar results were obtained using GPMVs generated from RPE1 cells (Figure S3), suggesting lipid peroxidation enhances phase separation in a cell-type-independent manner. For consistency, unless otherwise indicated, in all subsequent experiments, we utilized HeLa-cell-derived GPMVs.

Changes in the properties of the ordered or disordered domains are also predicted to alter the partitioning of lipid probes.^82^ We thus next quantified the effect of lipid peroxidation on the preference of the fluorescent domain markers for ordered versus disordered domains, defined as *P*_ordered_(81,83)


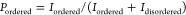
where *I*_ordered_ is the fluorescence intensity of the molecule of interest in the Lo domain and *I*_disordered_ is the fluorescence intensity in the Ld-like domain, with the underlying assumption that the fluorescence intensity is proportional to the concentration of fluorescent molecules in the two environments (Figure S4). *P*_ordered_ values range from 0 to 1, where *P*_ordered_ > 0.5 indicates that the marker prefers the ordered phase and *P*_ordered_ < 0.5 means that the domain marker prefers the disordered phase. We found that *P*_ordered_ increased for NBD-DSPE in response to lipid peroxidation, whereas *P*_ordered_ for DiD decreased (Figure 2C,D). These findings illustrate changes in the properties and compositions of the domains in response to lipid peroxidation.

We next asked whether lipid peroxidation impacts the relative abundance of ordered versus disordered domains. We measured the area fractions of Lo and Ld domains across multiple GPMVs and experiments (Figure 2E,F). Lipid peroxidation decreased the area fraction of Lo domains from ∼0.7 to ∼0.6 (Figure 2E,F), while the fraction of Ld domains significantly increased from ∼0.3 to ∼0.4 (Figure 2E,F). This suggests that either there is a shift in the total number of lipids present in the ordered phase versus the disordered phase or that the average area per molecule increases more strongly in the disordered than the ordered phase in response to lipid peroxidation.

The experiments described above were performed by directly subjecting GPMVs to conditions that induce lipid peroxidation. We next asked whether oxidative stress in living cells might give rise to similar effects. To test this, we treated HeLa cells with lipid peroxidation reagents for 30 min, then prepared GPMVs (Pre-LP, Figure 2G). The GPMVs from pretreated cells showed evidence for perturbed membrane phase behavior, including a significantly increased percentage of phase-separated GPMVs compared to control GPMVs prepared from untreated cells (Pre-LP versus Control, Figure 2G). These effects were not as dramatic as when GPMVs were directly treated with lipid peroxidation reagents (Post LP, Figure 2G), and were increased further by adding a post-treatment step (Pre + Post LP, Figure 2G). For simplicity, further experiments were carried out with GPMVs subjected directly to lipid peroxidation.

Taken together, these results suggest that lipid peroxidation has a profound effect on plasma membrane phase behavior, dramatically enhancing phase separation, altering the proportion of ordered and disordered domains, and modulating the partitioning of fluorescent lipids. To gain further insights into the effects of lipid peroxidation on the membrane, we next examined the lipid packing.

### Lipid Peroxidation Decreases Lipid Packing in Both Ordered and Disordered Domains

Lipid peroxidation generates products such as truncated lipids that perturb lipid packing in synthetic model membranes.^10,11,14−16,19,20^ To test whether similar effects are relevant in biological membranes, we turned to environmentally sensitive fluorescent probes that sense lipid packing. Specifically, we monitored the lifetime of the fluorescent reporter Di4 (Di-4-ANEPPDHQ),^94,95^ which has been widely shown to be dependent on membrane lipid packing, with lower Di4 lifetimes indicative of looser packing and more fluid membranes, and vice versa.^96^

**Figure 3:**
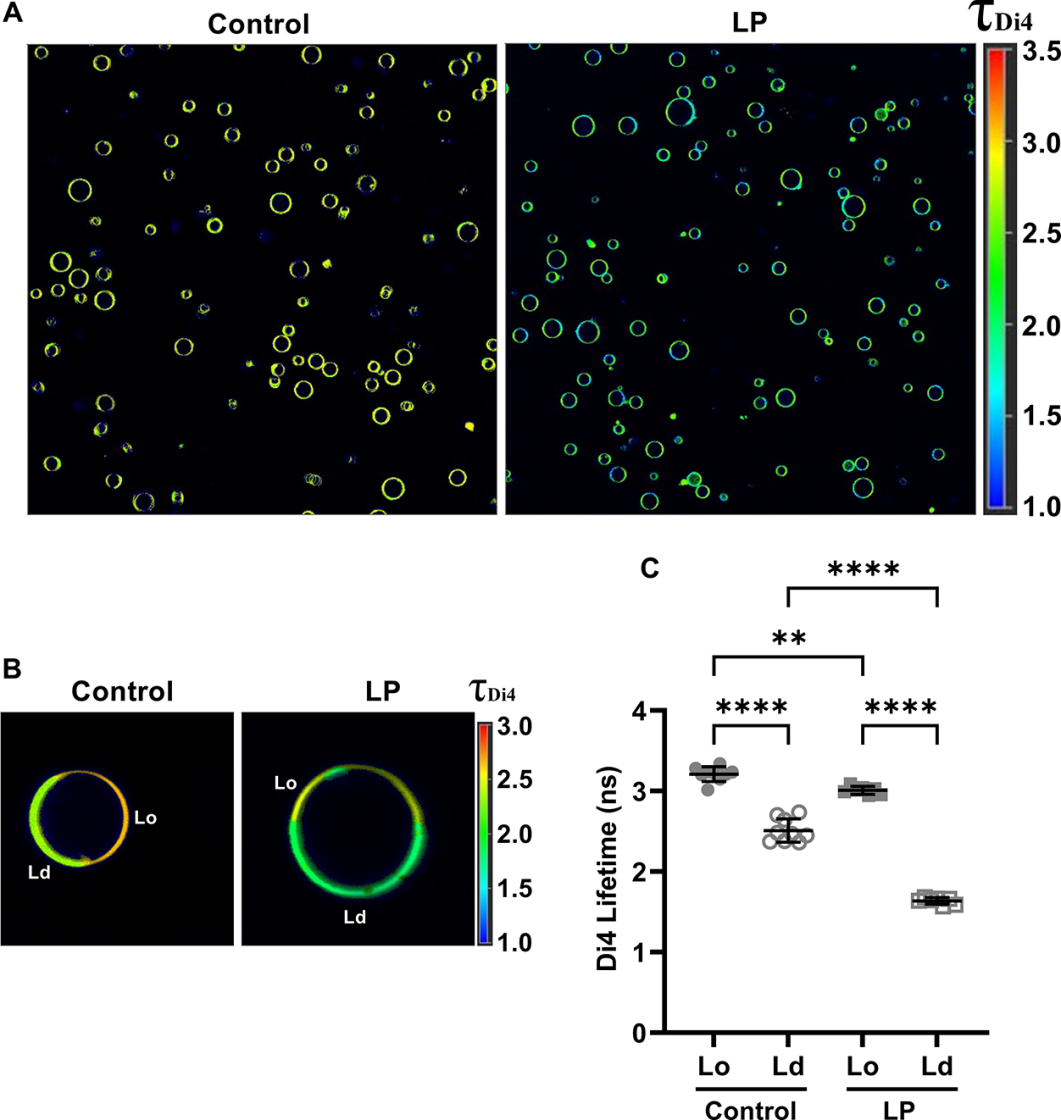
Lipid peroxidation decreases the level of lipid packing in both ordered and disordered domains. (A) Fluorescence lifetime imaging microscopy (FLIM) images of GPMVs labeled with Di4 under control conditions and following lipid peroxidation. Lookup table shows the Di4 lifetime (ns). (B) Representative FLIM images of individual GPMVs, highlighting differences in Di4 lifetime in Lo and Ld domains. Lookup table shows Di4 lifetime (ns). (C) Quantification of Di4 lifetimes in the Ld and Lo domains for control GPMVs and GPMVs subjected to lipid peroxidation. For the control sample, individual data points correspond to mean values for a field of >10–15 GPMVs taken from a single lifetime image; total number of control GPMVs = 104. For the LP sample, individual data points correspond to mean values for a field of >50 GPMVs taken from a single lifetime image; total number of LP GPMVs = 433. Error bars show mean ± SD. *P* values were determined by unpaired one-way ANOVA with Sidak’s multiple comparison test, α = 0.05 (95% confidence level), ****, *P* < 0.0001; n.s., not significant. Data are representative of two independent experiments.

Representative fluorescence lifetime imaging microscopy (FLIM) images of control and treated GPMVs are shown in Figure 3A. Based on the literature,^96^ domains with the higher lifetime correspond to the Lo domain and domains with the lower lifetime represent the Ld domain. Di4 lifetime decreased significantly in both domains in response to lipid peroxidation (Figure 3A,B). In Lo domains, the Di4 lifetime decreased from 3.26 ± 0.05 to 3.02 ± 0.07 ns following lipid peroxidation (Figure 3C). Lipid peroxidation also decreased Di4 lifetime in the disordered domains from 2.46 ± 0.09 to 1.62 ± 0.08 ns (Figure 3C). Notably, a larger change in Di4 lifetime was observed in Ld domains (Δτ = 0.84 ns) than in the Lo domain (Δτ = 0.24 ns) following lipid peroxidation, indicative of more dramatic disruption of lipid packing in Ld domains. This is likely due to the accumulation of higher levels of peroxidized lipids in Ld domains (Figure 1).

### Raft-Preferring Proteins Translocate to Disordered Domains in Response to Lipid Peroxidation

A major function of rafts in biological membranes is to laterally compartmentalize proteins.^46^ To facilitate sorting into or out of rafts, the structural features of proteins are tuned to match the fundamental properties of the membrane bilayer of each phase, including its thickness and lipid packing.^47,97,98^ Polyunsaturated lipids have been proposed to regulate the affinity of proteins for ordered domains in cells.^62,65,70,99−102^ However, the effect of peroxidation on the sorting of proteins into rafts is largely unknown. We thus set out to test whether the changes we observed in the properties of the ordered and disordered domains impact the partitioning of proteins thought to associate preferentially with either raft or non-raft domains in living cells.

**Figure 4:**
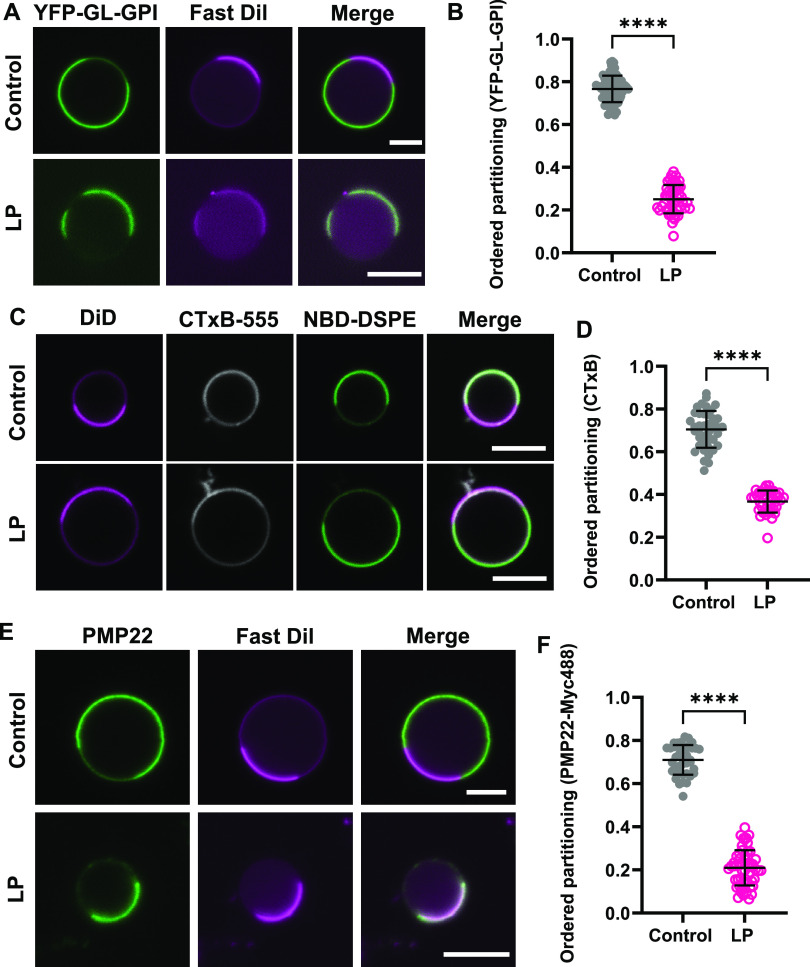
Raft-preferring proteins redistribute to disordered domains in response to lipid peroxidation. (A) Representative images of YFP-GL-GPI in HeLa-cell GPMVs. GPMVs were stained with Fast DiI after lipid peroxidation. (B) Impact of lipid peroxidation on ordered partitioning of YFP-GL-GPI. Each data point corresponds to individual GPMVs. Data are presented as mean ± SD for *n* = 47–68 GPMVs. (C) Representative images of CTxB-Alexa 555 labeling of COS-7 cell-derived GPMVs. After lipid peroxidation, GPMVs were sequentially labeled with CTxB-Alexa 555, NBD-DSPE, and DiD. (D) Impact of lipid peroxidation on the ordered partitioning of CTxB-Alexa 555. Data are presented as mean ± SD for 37–41 GPMVs. (E) Representative images of PMP22 in HeLa-cell GPMVs. GPMVs were labeled with an Alexa-488-labeled anti-myc antibody, subjected to lipid peroxidation, and then labeled with Fast DiI. (F) Impact of lipid peroxidation on ordered partitioning of PMP22. Data are presented as mean ± SD for 39–56 GPMVs. ****, *P* < 0.0001 for unpaired two-tailed *t* test. All data are representative of 2 independent experiments. Scale bars, 5 μm.

We first studied a GPI-anchored protein, YFP-GL-GPI.^83,85^ GPI-anchored proteins are normally targeted to rafts by the saturated acyl chains of the GPI anchor and are used extensively as markers for raft domains.^46,103^ As expected, YFP-GL-GPI partitioned into the ordered phase in untreated GPMVs (Figure 4). Lipid peroxidation dramatically disrupted this partitioning, leading instead to YFP-GL-GPI accumulation in disordered membrane regions marked by Fast DiI (Figure 4A). Similar results were obtained using several disordered-preferring reporter dyes, confirming that this shift was not due to changes in the localization of the marker dyes themselves (Figure S6A,B). The expulsion of YFP-GL-GPI from the more ordered phase was quantified as a dramatic decrease in *P*_ordered_ in response to lipid peroxidation (Figure 4B). Thus, peroxidation-induced changes disrupt the proper partitioning of the GPI anchor to ordered membrane domains.

To test the generality of these findings, we examined two other raft-associated proteins, cholera toxin B-subunit (CTxB) and Peripheral Myelin Protein 22 (PMP22). CTxB is a well-known raft marker used to study the functions and properties of raft domains.^104^ It binds up to five molecules of its glycolipid receptor, ganglioside GM1.^104^ Naturally occurring forms of GM1 are typically enriched in rafts in cells,^105^ and binding of CTxB to GM1 further enhances its association with raft domains via a clustering mechanism.^106^ In contrast, PMP22 is a multipass transmembrane protein.^107^ PMP22 also preferentially partitions in ordered domains in the plasma membrane, by mechanisms that are not yet clear.^81^ For our experiments, we utilized an *N*-glycosylation mutant of PMP22, N41Q-PMP22, that is targeted to the plasma membrane more efficiently than the wild-type form of the protein.^81^ N41Q-PMP22 (referred to hereafter as PMP22 for simplicity) contains a c-myc tag inserted into the second extracellular loop and can be visualized by labeling GPMVs with fluorescently labeled myc antibodies.^81^

To study the impact of lipid peroxidation on the phase preference of CTxB bound to endogenous GM1, we generated GPMVs from COS-7 cells, which bind high levels of CTxB.^106,108^ As expected, CTxB co-localized with the Lo domain marker NBD-DSPE in untreated GPMVs (Figure 4C). In contrast, when CTxB was added to GPMVs subjected to lipid peroxidation, it localized predominantly to Ld domains, corresponding to a significant decrease in *P*_ordered_ (Figure 4C,D). Similar results were obtained for PMP22 in HeLa-cell-derived GPMVs (Figure 4E,F).

Together, these results demonstrate that lipid peroxidation causes multiple classes of raft-preferring proteins, including a GPI-anchored protein, glycolipid-binding protein, and multipass transmembrane protein to shift out of the more ordered phase into the more disordered membrane environment. Thus, lipid peroxidation alters not only the lipid composition of both ordered and disordered domains but also their protein composition due to the mislocalization of raft proteins.

### Non-Raft Proteins Remain Associated with the Disordered Phase Following Lipid Peroxidation

We next examined the impact of lipid peroxidation on proteins that preferentially reside in non-raft domains in cells. Most examples of non-raft proteins are transmembrane proteins. As a test case, we studied the amyloid precursor protein (APP) and its cleavage product C99, key players in an amyloidogenic pathway linked to Alzheimer’s disease.^109^ In the amyloidogenic pathway, APP is cleaved by β-secretase to yield C99, which is subsequently processed by γ-secretase to generate amyloidogenic Aβ peptides.^110,111^ Although these processing events have long been thought to occur in raft-like environments,^112−114^ we recently found that C99-EGFP preferentially localizes in disordered regions of the plasma membrane.^83^ Lipid peroxidation has also been implicated in the progression of Alzheimer’s disease.^31,115−117^ We therefore wondered whether lipid peroxidation would enhance the partitioning of APP or C99 into ordered domains.

**Figure 5:**
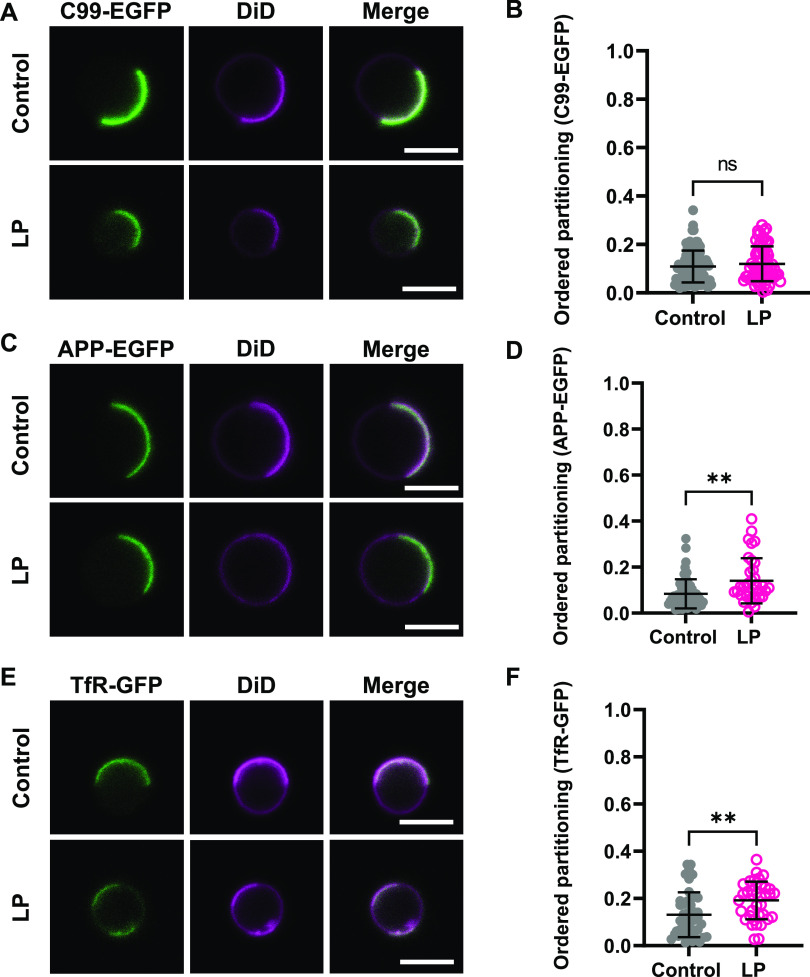
Non-raft proteins remain associated with disordered domains following lipid peroxidation. (A) Representative images of C99-EGFP in HeLa-cell GPMVs. (B) Quantification of ordered partitioning of C99-EGFP across multiple GPMVs. Each data point corresponds to an individual GPMV. Data are presented as mean ± SD for 65–102 GPMVs. (C) Representative images of APP-EGFP in HeLa-cell GPMVs. (D) Quantification of ordered partitioning of APP-EGFP across multiple GPMVs. Data are presented as mean ± SD for 38–54 GPMVs. (E) Representative images of TfR-GFP in HeLa-cell GPMVs. (F) Quantification of ordered partitioning of TfR-GFP across multiple GPMVs. Data are presented as mean ± SD for 35–46 GPMVs. **, *P* < 0.01 for unpaired two-tailed *t* test. All data are representative of 2 independent experiments. Scale bars, 5 μm.

To test this, we examined the phase preference of the GFP-tagged APP and C99 in GPMVs. To prevent APP and C99 from being cleaved by γ-secretase, we included the γ-secretase inhibitor DAPT throughout the experiment.^83^ Under control conditions, APP and C99 both co-localized with the DiD-enriched phase, corresponding to the more disordered region of the membrane (Figure 5A,C). Interestingly, both APP and C99-EGFP remained exclusively associated with disordered regions of the membrane following lipid peroxidation (Figure 5A–D). To investigate whether this is a general characteristic of non-raft proteins, we studied a GFP-tagged form of transferrin receptor, TfR-GFP.^118^ TfR-GFP localized primarily in disordered domains under control conditions, as expected (Figure 5E) and remained enriched in the more disordered regions of the membrane following lipid peroxidation (Figure 5E,F). Together, these findings suggest that non-raft proteins continue to partition in this environment in peroxidized membranes.

## Discussion

**Figure 6:**
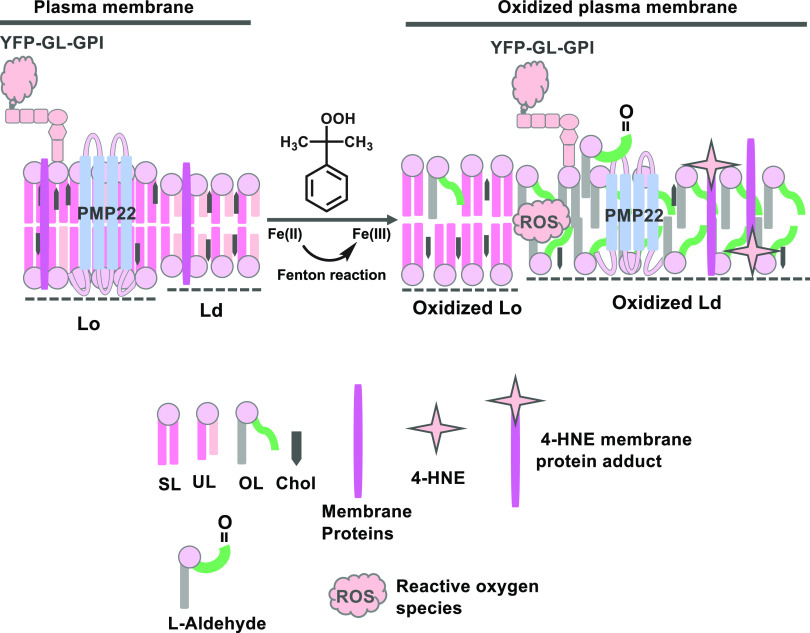
Working model for how lipid peroxidation induced via the Fenton reaction impacts ordered and disordered domains in biological membranes. Peroxidized lipids and their bioactive products such as 4-HNE preferentially accumulate in disordered domains. This is accompanied by increases in the relative abundance of the disordered phase, decreased lipid packing in both phases, and changes in protein composition in both phases as the result of the selective redistribution of proteins from the ordered to the disordered phase. SL, saturated lipid; UL, unsaturated lipid; OL, oxidized lipid; chol, cholesterol.

In the current study, we used GPMVs as a model to investigate how lipid peroxidation driven by the Fenton reaction impacts the properties of membrane rafts in biological membranes. Our findings reveal that lipid peroxidation significantly impacts several aspects of raft homeostasis (Figure 6).

One of the most striking effects of lipid peroxidation is the dramatic enhancement of membrane demixing into coexisting ordered and disordered domains, resulting in nearly all vesicles becoming phase-separated. To the best of our knowledge, this represents among the largest responses to perturbation ever reported in the GPMV model. Several factors likely contribute to the heightened propensity of the membrane to phase-separate. The most obvious is the accumulation of peroxidized lipids and their products in the disordered phase. This finding is in some ways not unexpected given that non-raft domains are generally thought to be enriched in lipids with unsaturated chains, including PUFAs. Furthermore, we observed a significantly increased area fraction of the disordered phase and decreased lipid packing therein in peroxidized GPMVs. We also detected evidence for covalent modifications of membrane proteins and their segregation into disordered domains by the bioactive aldehyde 4-HNE. The presence of this and other byproducts of lipid oxidation could also contribute to disruptions in membrane structure and decreased lipid packing.

Importantly, changes in membrane properties following lipid peroxidation were not limited to disordered domains: peroxidized lipids and decreased lipid packing were also observed in the more ordered phase. Although this result may seem surprising, it is consistent with reports that some PUFAs can be incorporated in rafts.^64,119^ Other lipid components of ordered domains may serve to dampen the progression of lipid peroxidation. For example, sphingomyelin has been reported to inhibit oxidative damage by preventing propagation of lipid peroxidation,^120,121^ and enhanced levels of cholesterol have been reported to reduce membrane peroxidation in fibroblasts from patients with familial Alzheimer’s disease and to suppress lipid peroxidation in tumor cells.^122,123^ In future studies, it will be important to elucidate how the effects of lipid peroxidation depend on factors that influence membrane lipid composition, such as growth conditions, cell cycle stage, nutritional sources, and disease state.

We also found that multiple proteins, including CTxB, GPI-anchored proteins, and multipass transmembrane proteins, were dislodged from ordered into disordered domains in response to lipid peroxidation. In contrast, non-raft proteins remained localized correctly despite the extensive accumulation of the peroxidized lipids in the more disordered phase. Thus, lipid peroxidation causes a loss of segregation of raft from non-raft proteins. The finding that raft proteins are selectively mislocalized implies that their affinity for ordered phases is especially sensitive to changes in membrane structure that occur in response to lipid peroxidation. The exact mechanism underlying this sensitivity is not yet clear, but could be linked to loss of PUFAs and/or changes in the local structure of ordered domains, both of which have been suggested to aid in protein sorting.^69,70,124^ Changes in protein structure induced by covalent modifications by bioactive products of peroxidation^21−23^ could also contribute to the selective loss of protein affinity for the more ordered phase. In intact cells, more indirect mechanisms may also play a role.^125^

It is important to note that two of the three raft proteins that we studied are coupled to the membrane through lipids. This raises the question of whether changes in their partitioning are due to chemical changes in their lipid anchors or reflect more general changes in the membrane environment. Most cell surface-associated GPI-anchored proteins are thought to contain two saturated fatty acids^126^ which presumably are not chemically modified in response to oxidative stress. Ganglioside GM1, the high-affinity receptor for CTxB, is also generally regarded to be a raft-preferring lipid.^105^ On the other hand, the association of both GPI-anchored proteins and CTxB with rafts is known to depend on specific properties of Lo domains.^127,128^ Thus, it seems likely that oxidation-induced changes in ordered domains drive their redistribution into a more disordered phase. Interestingly, in contrast to the behavior of raft proteins, the partitioning of reporter dye NBD-DSPE into Lo domains was enhanced rather than reversed in oxidized GPMVs. We speculate that this enhancement is primarily driven by the increased difference in lipid packing between the two phases following lipid peroxidation as opposed to the more nuanced dependence of raft protein partitioning on additional chemical and physical features of each phase.

How widespread the consequences of the oxidation-dependent displacement of proteins from ordered domains are on cellular structure and function remains to be determined. This could be especially important for signaling pathways that are regulated by raft formation, raft targeting mechanisms, or segregation of raft and non-raft proteins.^129,130^ Another critical question is how the structure and function of individual membrane proteins are impacted by oxidized lipids. Evidence is already beginning to accumulate that oxidized lipids modulate the activity of transmembrane proteins whose function is sensitive to their membrane environment, such as G-protein-coupled receptors (GPCRs).^131^ Such effects could be important not only for proteins that normally prefer to function in an ordered lipid environment but also for those that normally operate in disordered domains.

Lipid peroxidation can be initiated by a variety of mechanisms.^6^ In the current study, we used a nonenzymatic approach to generate reactive oxygen species driven by the Fenton reaction. The Fenton reaction occurs in biological systems and is thought to contribute to ferroptosis, a form of cell death triggered by iron-dependent lipid peroxidation.^25,26^ Importantly, recent evidence suggests that plasma membrane lipids are among those that undergo peroxidation during ferroptosis.^132,133^ Thus, disturbances in plasma membrane raft homeostasis are likely to be among the cellular defects that occur as ferroptosis is initiated and executed. PUFAs are also susceptible to react with reactive oxygen species which are produced by enzymatic (e.g., 12/15 lipoxygenase) processes, as well as those produced by photosensitizers and other mechanisms.^23,134^ An important goal for the future will be to establish whether these differing peroxidation mechanisms, including those observed under both physiological and pathophysiological conditions, have similar or distinct effects on raft homeostasis.

## Conclusions

In conclusion, our study illustrates that lipid peroxidation has profound effects on both liquid-ordered and liquid-disordered domains in biological membranes including their stability, abundance, and lipid and protein composition. These findings suggest that disruptions of membrane phase behavior may play a previously unrecognized role in ferroptosis and contribute to the pathology and progression of diseases linked to oxidative stress. Ultimately, understanding the consequences of lipid peroxidation on cellular membranes and their organization may offer new avenues to therapeutically target or exploit oxidative stress in various disease contexts.

## Supporting Information Available

Experimental Section and figures showing that both Fe(II) and cumene hydroperoxide are required for lipid peroxidation to occur in GPMVs, representative control experiments for 4-HNE staining, an example of how fluorescence intensity was quantified to calculate *P*_ordered_, lipid peroxidation enhances phase separation in RPE1-derived GPMVs, and lipid peroxidation causes the translocation of YFP-GL-GPI from ordered to disordered domains (PDF)

## Abbreviations

4-HNE: 4-hydroxynonenal
APP: amyloid precursor protein
CTxB: cholera toxin B subunit
DiD: 1,1′-dioctadecyl-3,3,3′,3′-tetramethylindodicarbocyanine perchlorate
Di4: Di-4-ANEPPDHQ
FLIM: fluorescence lifetime imaging microscopy
GPMV: giant plasma membrane vesicle
*I*_disordered_: fluorescence intensity of a given fluorescent dye or protein in the disordered phase
*I*_ordered_: fluorescence intensity of a given fluorescent dye or protein in the ordered phase
Ld: liquid-disordered
Lo: liquid-ordered
NBD-DSPE: 1,2-distearoyl-*sn*-glycero-3-phosphoethanolamine-*N*-(7-nitro-2–1,3-benzoxadiazol-4-yl)
PMP22: Peripheral Myelin Protein 22
*P*_ordered_: ordered phase partitioning fraction
PUFA: polyunsaturated fatty acid
TfR: transferrin receptor
