# Supplementary material for: Lipid Peroxidation Drives Liquid–Liquid Phase Separation and Disrupts Raft Protein Partitioning in Biological Membranes
Source: J Am Chem Soc. 2024 Jan 3;146(2):1374–87. doi: 10.1021/jacs.3c10132 (PMC10797634; doi:10.1021/jacs.3c10132)
Supplement: Supplementary file 1 — ja3c10132_si_001.pdf [file ja3c10132_si_001.pdf]

Supporting Material

Lipid peroxidation drives liquid-liquid phase separation and disrupts raft protein partitioning in biological membranes

Muthuraj Balakrishnan[1,2] and Anne K. Kenworthy[*,1,2]

[1]Center for Membrane and Cell Physiology, University of Virginia, Charlottesville, VA, 22903, USA.

[2]Department of Molecular Physiology and Biological Physics, University of Virginia School of Medicine, Charlottesville, VA, 22903, USA

*To whom correspondence should be addressed. Akk7hp@virginia.edu

Experimental Section

Safety

No unexpected, new, and/or significant hazards or risks are associated with the reported work.

Materials

DTT was purchased from Research Products International (Mount Prospect, USA) and was made fresh for every experiment. Paraformaldehyde was purchased as a solid from Fisher Scientific (catalog # AC416780250) and prepared as a 4% stock solution in H_2O. All other chemicals such as $CaCl_2$, HEPES, NaCl, Cumene hydroperoxide (catalog # 247502), and Iron (II) perchlorate hydrate 98% (catalog # 334081-5G) was purchased from Sigma. 18:0 NBD-PE (NBD-DSPE) 1,2-distearoyl-sn-glycero-3-phosphoethanolamine-N-(7-nitro-2-1,3-benzoxadiazol-4-yl) (ammonium salt) was purchased from Avanti Polar Lipids (catalog # 810141). DiD' oil (catalog # D307), DiIC18(5) (catalog # D12730), BODIPY 581/591 C11 (catalog # D3861), and Di-4-ANEPPDHQ (Di4) (catalog # D36802) were purchased from Thermo Fisher Scientific. DiIC12 (1,1′-didodecyl-3,3,3′,3′-tetramethylindocarbocyanine perchlorate) (catalog # D383) and Fast DiI (catalog # D7756) were purchased from Invitrogen (Life Technologies). DAPT was obtained from Selleckchem (catalog # S2215). Cumene hydroperoxide and iron II perchlorate were each prepared as 10 mM stock solutions in H_2O and stored at 4° C.

Antibodies used in this study include anti-myc Alexa Fluor 488 conjugate mAb (mouse) (Cell Signaling Technology, (catalog #2279S), rabbit anti-4 hydroxynonenal antibody (Abcam, catalog #ab46545), and Alexa Fluor 488 donkey anti-rabbit IgG (H+L) (Invitrogen, catalog #A21206). Cholera toxin subunit B (recombinant), Alexa Fluor 555 conjugate was from Themo Fisher Scientific (catalog #C22843).

cDNA constructs used in this study include YFP-GL-GPI (model GPI-anchored protein; raft marker) [1]; TfR-GFP (transferrin receptor; non-raft marker), a gift from Dr. Ilya Levental [2]), and N41Q PMP22 (raft-associated multi pass transmembrane protein) [3]. C99-EGFP was as previously described [4] and was a gift from Dr Paola Pizzo [5]. APP-GFP was also a gift from Dr Paola Pizzo [5].

Cell culture

WT-HeLa, COS-7, and RPE-1 cells were acquired from ATCC (Manassas, USA). Cells were cultured in Dulbecco's modified Eagle medium (DMEM) containing 10% fetal bovine serum (FBS) and 1% pen/strep at 37 °C and 5% CO_2. For all studies, cells were freshly plated a day prior to the preparation of GPMVs such that they would be 75-85% confluent on the day of the experiment.

Transfection

For experiments using transfected cells, approximately 24 h prior to transfection, cells were plated to be 40–50% confluent at the time of transfection. Cells were transfected using FuGene-HD Transfection reagent (Promega, catalog #E2311) with a FuGene: DNA ratio of 3:1 using 4

μg of DNA in OptiMEM Reduced Serum Medium (ThermoFisher Scientific Catalog #11058021). The transfection medium was removed from cells ~6–8 h post transfection, and cells were washed with Dulbecco's modified Eagle medium and fresh culture media was added to each plate. GPMVs were prepared 24 h post transfection.

For experiments using C99-EGFP or APP-EGFP, twenty-four hours after transfection, cell culture media was exchanged for fresh media supplemented with 20 μM DAPT. DAPT was also included in all buffers used for GPMV preparation.

Preparation and imaging of GPMVs

GPMVs were prepared as previously described [6]. In brief, cells were washed twice with GPMV buffer (2 mM $CaCl_2$ /10 mM HEPES /0.15M NaCl, pH 7.4), followed by two washes with GPMV active buffer (GPMV buffer plus 25 mM formaldehyde and 2 mM DTT). Cells were then incubated with GPMV active buffer for 2 h at 37º C with shaking at 95-100 RPM. The GPMV-containing supernatant was decanted into a centrifuge tube and allowed to settle for 1 h at RT or overnight at 4º C. Aliquots of GPMVs (100 μL) were transferred from the bottom of the tube to a fresh tube for labeling or lipid peroxidation. We observed no difference in GPMV quality whether we imaged immediately or 24 h post-GPMV preparation.

To perform lipid peroxidation experiments, 100 μL of GPMVs were treated with a combination of 500 μM cumene hydroperoxide and 50 μM iron (II) perchlorate to serve as a source of ROS. GPMVs were post labelled with 1 μg/ml DiD (Ld phase marker [7, 8], diluted from a 1mg/mL stock in ethanol) to mark this disordered phase and 0.5 μg/ml NBD-DSPE (Lo phase marker, diluted from a 1mg/mL stock in ethanol) to mark the ordered phase. 30 minutes prior to imaging, 100 μL of GPMV solution was sandwiched between two coverslips coated with bovine serum albumin (BSA: 1mg/mL solution stock solution).

For some experiments, GPMVs were prepared from cells subjected to lipid peroxidation. Here, cells were incubated in the presence of 500 μM cumene hydroperoxide and 50 μM iron (II) perchlorate for 30 min and washed prior to GPMV preparation. A subset of GPMVs were then further incubated with 500 μM cumene hydroperoxide and 50 μM iron (II) perchlorate prior to labeling and imaging as described above.

GPMVs were imaged at RT using a Zeiss LSM 880 confocal microscope using a 63X oil objective. For most experiments, the confocal pinhole was set to 63.1 nm for all the channel and gain set to 700 for three different channels. Fluorophores were excited using the 488 nm line of an argon laser (NBD-DSPE), the 540 nm line of a HeNe laser (BODIPY 581/591) and the 633 nm line of a HeNe laser (DiD). Images were collected at a 1X digital zoom with a 1024 × 1024 or 512 × 512-pixel resolution for all the measurements and at 8–10X digital zoom for quantifying phase partitioning with a 1024 × 1024 or 512 × 512-pixel resolution.

Detection of lipid peroxidation

The lipid peroxidation sensor C11-BODIPY (581/591) (Invitrogen, catalog # D3861) used to detect lipid peroxidation. For these experiments, GPMVs were first subjected to lipid peroxidation with 500 μM cumene hydroperoxide and 50 μM iron (II) perchlorate at RT or left

untreated. They were then labeled with 1 μM of C11-BODIPY (581/591) and allowed to settle for 15-20 min prior to imaging at RT by confocal microscopy. For some experiments, GPMVs were labeled with C11-BODIPY (581/591), immediately followed by DiD.

Di4 fluorescence lifetime imaging

GPMVs were prepared from HeLa cells as described above. They were then either left untreated or incubated with 500 μM cumene hydroperoxide and 50 μM iron (II) perchlorate. Di4 was added to a final concentration of 1 μg/ml from a stock solution of 1 mg/mL Di4 in ethanol. The GPMVs were then sandwiched between two coverslips coated with 1 mg/mL BSA solution and allowed to incubate for an additional 20 min prior to lifetime measurements.

Di4 lifetime images were performed on a Leica TCS SP8 system consisting of a Leica DMi8 inverted scanning confocal microscope with time-correlated single photon counting (TC-SPC) system and an 80 MHz-pulsed supercontinuum white light laser which allows continuously tunable excitation in the visible range coupled with filter-free detection. Samples were imaged using an HC PL APO CS2 63X/1.20 water objective. Di4 was excited at 488 nm and the emission was collected in the 550-800 nm range. The photon count rate was kept under 0.5 photons per laser pulse by adjusting the laser power, and sufficient frames were cumulatively acquired to obtain at least 100 photons per pixel. The fluorescence decay curves were fit with a bi-exponential re-convolution function adjusted to the instrument response function, and the average intensity-weighted lifetime was calculated for individual vesicles or area of interest and represented in the FLIM images as Di4 lifetime. Data is reported from two independent experiments. Statistical analyses were performed using GraphPad Prism 9.3.0.

Immunofluorescence labeling of 4-HNE and PMP22

For 4-HNE labeling, GPMVs were prepared from HeLa cells as described above. Anti-4 hydroxynonenal antibody (Abcam, catalog # ab46545) was added to the GPMVs solution at a 1:500 dilution and gently agitated in the dark at RT for 1-2 h. GPMVs were subsequently labelled with a 1:500 dilution of Alexa Fluor 488 Donkey anti-Rabbit IgG (H+L) (Invitrogen, catalog # A21206) and gently agitated in the dark at RT for a minimum of 2-3 h. The GPMV preparation was split in half. Half of the labeled GPMVs were left untreated. The other half were incubated with a combination of 500 μM cumene hydroperoxide and 50 μM iron (II) perchlorate for 30 minutes. Finally, DiD was added to a final concentration of 1 μg/ml from a stock solution of 1 mg/mL in ethanol. The labeled GPMVs were then sandwiched between two coverslips coated with 1mg/mL BSA separated by a 1 mm silicon spacer and allowed to settle for 30 min at RT prior to imaging. As controls, either the primary antibody or secondary antibody was excluded from the labeling reactions.

For PMP22 labeling, GPMVs derived from HeLa cells transiently expressing PMP22 were then decanted into a 6 well plate. Alexa 488 mouse anti-myc mAb (Cell Signaling Technology, catalog # 2279S) was added to the solution at a 1:500 dilution and gently agitated in the dark at RT for at 3-5 h. GPMVs were then collected, transferred to a fresh tube, and allowed to settle for 1-2 h at RT. Next, GPMVs were either left untreated or subjected to lipid peroxidation as described above, then labelled at RT with 0.5 μg/ml Fast DiI (diluted from a 0.5 mg/mL stock in

ethanol) to mark the disordered phase. GPMVs were mounted for imaging as described above. Imaging of GPMVs containing PMP22 was performed 4-6 h after labeling.

CTxB labeling

GPMVs were prepared from COS-7 cells and either left untreated or subjected to lipid peroxidation using the procedures described above for HeLa cells GPMVs. Alexa Fluor 555-cholera toxin subunit B (Invitrogen, catalog # C22843) was added to 100 µl of GPMVs at a 1:100 dilution of a 1 mg/ml stock. The GPMVs were post labelled with 0.5 µg/ml NBD-DSPE (diluted from a 1mg/mL stock in ethanol) and 1 µg/ml DiD (diluted from a 1 mg/mL stock in ethanol) to mark the ordered and disordered phases, respectively, mounted, and imaged as described above.

Quantification of Phase Partitioning

$P_{ordered}$ was calculated as previously described [6, 9, 10]:

$$P_{ordered} = I_{ordered}/I_{ordered} + I_{disordered}$$

where $I_{ordered}$ and $I_{disordered}$ are the fluorescence intensity of a given fluorescent dye or protein in the ordered and disordered phases, respectively. To determine the phase of each domain, GPMVs were labeled with either an ordered membrane phase marker (NBD-DSPE), a disordered membrane phase marker (DiD), or both. ImageJ was used to manually perform a line scan across individual GPMVs to determine the highest fluorescent intensity at each pixel. The line scans were smoothed using a moving average (5 to 10 pixels) in Excel then the peak values were used to calculate $P_{ordered}$ values. A $P_{ordered} > 0.5$ indicates a preference for the more ordered phase, and $P_{ordered} < 0.5$ corresponds to a disordered phase preference.

Calculation of percentage of phase separated vesicles

Where indicated, the percentage of phase separated vesicles was calculated from tiled images using a semi-automated Matlab-based program, VesA [10]. Data from the green channel was used for the analysis.

Statistical analysis

GraphPad Prism 9 was used to carry out statistical analyses.

References

1. Keller, P.; Toomre, D.; Diaz, E.; White, J.; Simons, K., Multicolour imaging of post-Golgi sorting and trafficking in live cells. *Nat. Cell Biol.* **2001,** *3*, 140-149.

2. Zhou, Y.; Maxwell, K. N.; Sezgin, E.; Lu, M.; Liang, H.; Hancock, J. F.; Dial, E. J.; Lichtenberger, L. M.; Levental, I., Bile acids modulate signaling by functional perturbation of plasma membrane domains. *J Biol Chem* **2013,** *288* (50), 35660-70.

3. Marinko, J. T.; Kenworthy, A. K.; Sanders, C. R., Peripheral myelin protein 22 preferentially partitions into ordered phase membrane domains. *Proc Natl Acad Sci U S A* **2020,** *117* (25), 14168-14177.

4. Capone, R.; Tiwari, A.; Hadziselimovic, A.; Peskova, Y.; Hutchison, J. M.; Sanders, C. R.; Kenworthy, A. K., The C99 domain of the amyloid precursor protein resides in the disordered membrane phase. *J Biol Chem* **2021,** *296*, 100652.

5. Florean, C.; Zampese, E.; Zanese, M.; Brunello, L.; Ichas, F.; De Giorgi, F.; Pizzo, P., High content analysis of gamma-secretase activity reveals variable dominance of presenilin mutations linked to familial Alzheimer's disease. *Biochim Biophys Acta* **2008,** *1783* (8), 1551-60.

6. Sezgin, E.; Kaiser, H. J.; Baumgart, T.; Schwille, P.; Simons, K.; Levental, I., Elucidating membrane structure and protein behavior using giant plasma membrane vesicles. *Nat Protoc* **2012,** *7* (6), 1042-51.

7. Sezgin, E.; Levental, I.; Grzybek, M.; Schwarzmann, G.; Mueller, V.; Honigmann, A.; Belov, V. N.; Eggeling, C.; Coskun, U.; Simons, K.; Schwille, P., Partitioning, diffusion, and ligand binding of raft lipid analogs in model and cellular plasma membranes. *Biochim Biophys Acta* **2012,** *1818* (7), 1777-84.

8. Klymchenko, A. S.; Kreder, R., Fluorescent probes for lipid rafts: from model membranes to living cells. *Chem Biol* **2014,** *21* (1), 97-113.

9. Raghunathan, K.; Wong, T. H.; Chinnapen, D. J.; Lencer, W. I.; Jobling, M. G.; Kenworthy, A. K., Glycolipid crosslinking is required for cholera toxin to partition into and stabilize ordered domains. *Biophys J* **2016,** *111* (12), 2547-2550.

10. Fricke, N.; Raghunathan, K.; Tiwari, A.; Stefanski, K. M.; Balakrishnan, M.; Waterson, A. G.; Capone, R.; Huang, H.; Sanders, C. R.; Bauer, J. A.; Kenworthy, A. K., High-content imaging platform to discover chemical modulators of plasma membrane rafts. *ACS Cent Sci* **2022,** *8* (3), 370-378.

Figure S1.

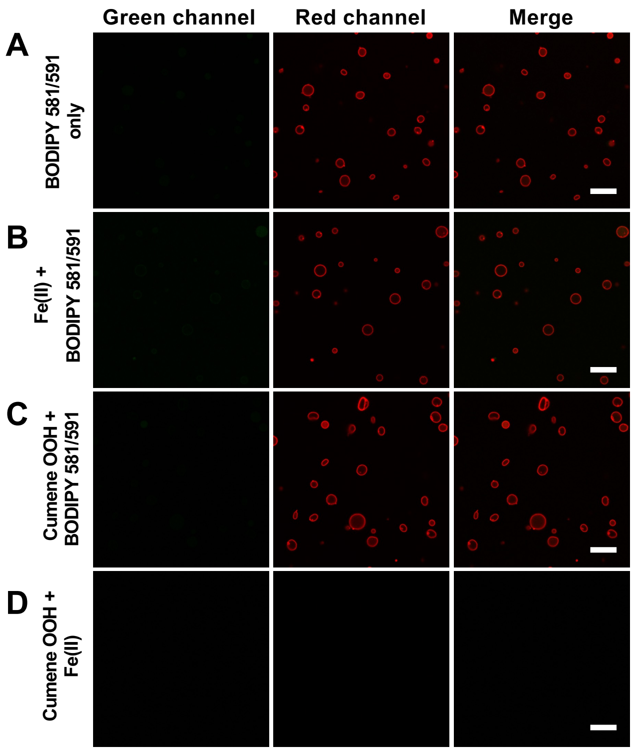

Figure S1. Both Fe (II) and cumene hydroperoxide are required for lipid peroxidation to occur in GPMVs. HeLa cell-derived GPMVs were incubated with the following reagents prior to imaging: (**A**) BODIPY 581/591 (1 μM); (**B**) Fe(II) (50 μM) followed by BODIPY 581/591 (1 μM); (**C**) Cumene hydroperoxide (500 μM) followed by BODIPY 581/591 (1 μM); and (**D**) Fe(II) (50 μM) and cumene hydroperoxide (500 μM) in the absence of BODIPY 581/591. No fluorescence was observed in the green channel when one or both lipid peroxidation reagents were omitted from the reaction, and no fluorescence was detected in either the green or red channels in the absence of BODIPY 581/591. Scale bar, 20 μm.

Figure S2.

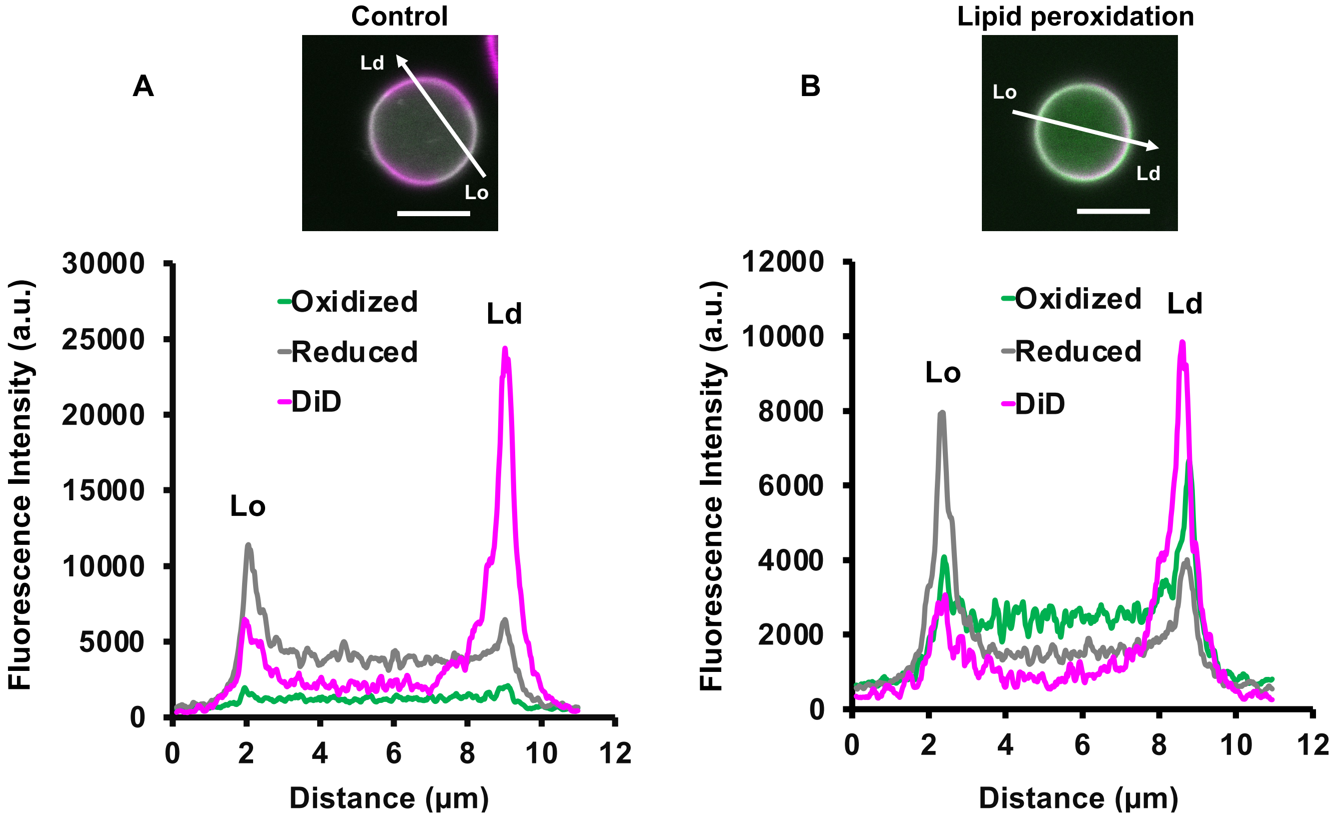

Figure S2. Example of how fluorescence intensity was quantified to calculate the ratio of oxidized and reduced forms of BODIPY 581/591 in each phase. Representative examples are shown for (**A**) a control GPMV and (**B**) a GPMV subjected to lipid peroxidation. A line bisecting each GPMVs was manually positioned to pass through both the Ld domain (enriched in DiD) and Lo domain (depleted in DiD). Plots of the fluorescence intensity along the line for all three fluorescence channels was generated. The peaks of the line plots were used to quantify the ratio of oxidized and reduced forms of BODIPY 581/591 in each phase as described in the Materials and Methods. a.u., arbitrary units. Scale bars, 5 µm.

Figure S3.

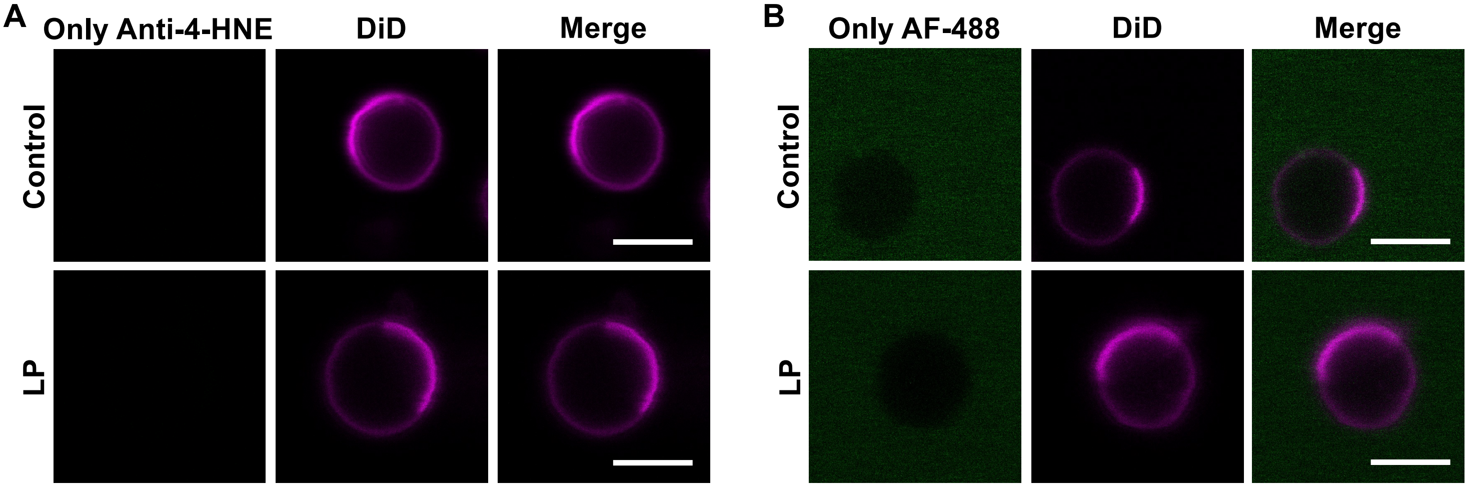

Figure S3. Representative control experiments for 4-HNE staining. GPMVs were either left untreated or subjected to lipid peroxidation, immunolabeled with an anti-4-HNE antibody or Alexa-488 secondary antibody, and then stained using DiD. Examples of representative GPMVs are shown. **(A)** Control verifying lack of fluorescent antibody staining in the absence of secondary antibody. **(B)** Control verifying lack of fluorescent antibody staining in the absence of primary antibody. Scale bars, 5 μm.

Figure S4.

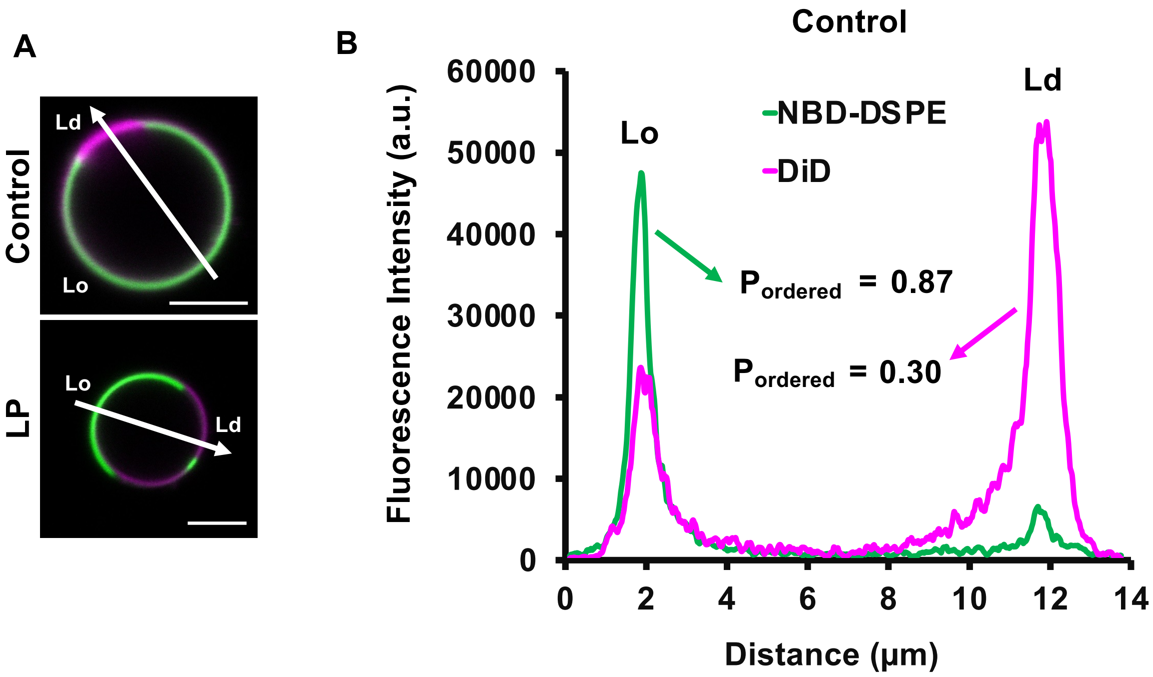

C

$$P_{ordered} = \frac{I_{ordered}}{I_{ordered} + I_{disordered}}$$

D

$P_{ordered} > 0.5$ **Ordered phase**

$P_{ordered} < 0.5$ **Disordered phase**

$P_{ordered} = 0.5$ **No phase preference**

Figure S4. Example of how fluorescence intensity was quantified to calculate $P_{ordered}$. (**A**) Representative examples are shown for a control GPMV and a GPMV subjected to lipid peroxidation. A line bisecting each GPMVs was manually positioned to pass through both the Ld domain (enriched in DiD) and Lo domain (enriched in NBD-DSPE). Scale bars, 5 µm. (**B**) Plots of the fluorescence intensity along the line for both fluorescence channels was generated. The peaks of the line plots were used to quantify $I_{ordered}$ and $I_{disordered}$ for each probe. a.u., arbitrary units. (**C**) Equation used to calculate $P_{ordered.}$ (**D**) Definitions of $P_{ordered}$ values.

Figure S5.

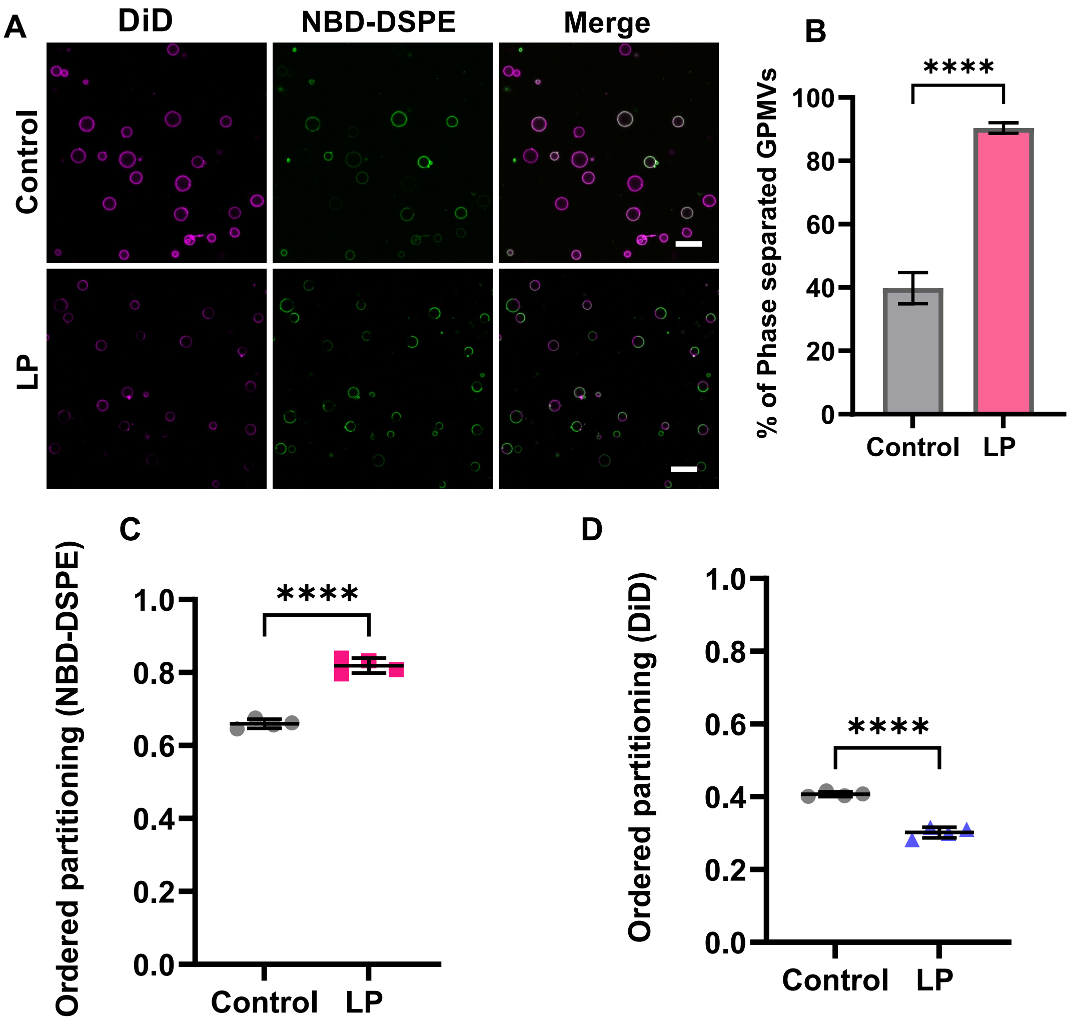

Figure S5. Lipid peroxidation enhances phase separation in RPE1-derived GPMVs. (**A**) GPMVs were either left untreated (control) or subjected to lipid peroxidation (LP). They were then labeled sequentially with NBD-DSPE (green) and DiD (magenta) prior to imaging at RT using confocal microscopy. Scale bar, 20 µm. (**B**) Quantification of the percentage of phase separated GPMVs for control versus lipid peroxidation conditions. The % of phase separated GPMVs was calculated using the green channel using VesA software. Bars correspond to mean ± SD for >100 GPMVs per condition. (**C, D**) Impact of lipid peroxidation on ordered partitioning of Lo (NBD-DSPE) and Ld (DiD) reporter dyes. Points in C and D represent >50 GPMVs in each group. ****, $P < 0.0001$ using unpaired two-tailed t-test.

Figure S6

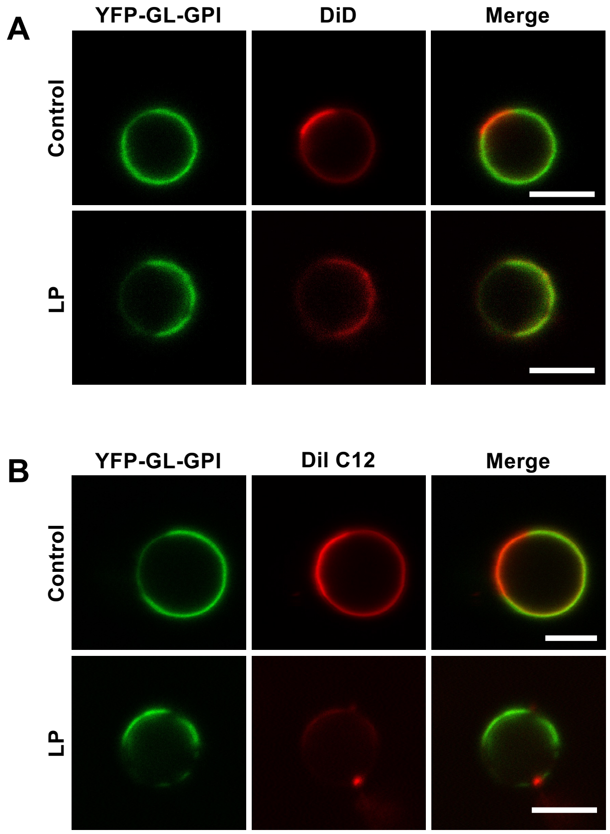

Figure S6. Lipid peroxidation causes the translocation of YFP-GL-GPI from ordered to disordered domains. Representative images of YFP-GL-GPI in HeLa cell-derived GPMVs under control conditions and following lipid peroxidation. GPMVs were labeled with the disordered domain markers (**A**) DiD or (**B**) DiIC12 after lipid peroxidation. Scale bars, 5 μm.
